# The Magnitude of the Secondary B Cell Response is Shaped by Pre‐Existing Antibodies Rather Than the Number of Memory B Cells Present

**DOI:** 10.1002/eji.70315

**Published:** 2026-11-11

**Authors:** Malte J. Hoormann, Noémi Becza, Lingling Yao, Anton V. Gorbachev, Stefanie Kuerten, Magdalena Tary‐Lehmann, Greg A. Kirchenbaum, Paul V. Lehmann

**Affiliations:** ^1^ Research and Development Cellular Technology Ltd. (CTL) Shaker Heights Ohio USA; ^2^ University of Bonn and University Hospital Bonn Institute of Neuroanatomy Bonn Germany; ^3^ Contract Laboratory CTL Shaker Heights Ohio USA

**Keywords:** clonal expansion, ELISPOT, FluoroSpot, humoral, Ig class, immune monitoring, immunological memory, memory B cell, plasma cell, vaccination

## Abstract

Clonal expansion of memory lymphocytes after each antigen encounter is the primary mechanism for amplifying immunity. For most vaccines, boosters are common practice and are expected to stimulate proliferation of pre‐existing memory B cells (B_mem_), thereby expanding the antigen‐specific B_mem_ pool, along with driving their differentiation into antibody‐producing plasma cells that replenish antibody titers. It is widely assumed that the number of B_mem_ present in the body prior to administration of a booster vaccination will define the magnitude of the ensuing response. By comparing B_mem_ frequencies and antibody titers in the same individuals after primary and secondary vaccination with SARS‐CoV‐2 Spike (S‐antigen)‐encoding mRNA, we found that expansion of B_mem_ and the relative increase of the secondary antibody response were not determined by the number of B_mem_ measured before the second vaccine inoculation. Instead, both were inversely correlated with levels of S‐antigen‐specific serum IgG prior to the secondary antigen exposure. Collectively, these data suggest that secondary B cell responses are constrained by antibody feedback inhibition of B_mem_, rather than their scarcity.

## Graphical abstract

Booster vaccinations are assumed to trigger stereotypical re‐expansions of memory B cells (B_mem_). In a SARS‐CoV‐2 prime‐boost cohort, however, the magnitude of the secondary response was not predicted by preboost B_mem_ frequencies, but was inversely associated with pre‐existing specific antibody levels, pointing to antibody feedback as the dominant limiting parameter.

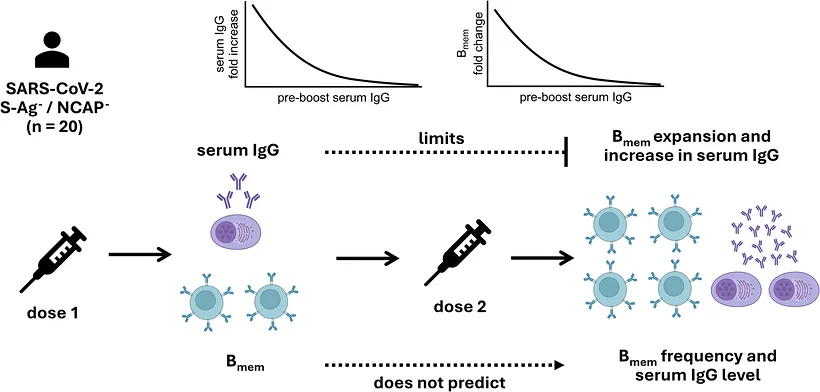

**Abbreviations**
ASC: antibody‐secreting cell
B_mem_
: memory B cell
CM: complete medium
ELISA: enzyme‐linked immunosorbent assay
ELISPOT: enzyme‐linked immunosorbent spot assay
FC: flow cytometry
NCAP: nucleocapsid protein
PBMC: peripheral blood mononuclear cell
PBS: phosphate‐buffered saline
PC: plasma cell
S‐antigen: SARS‐CoV‐2 Spike
SFU: spot‐forming unit

## 1 Introduction

Humoral immunity is mediated by the B cell system. When a primary B cell response is engaged after natural infection or the first administration of a vaccine, naïve antigen‐specific B cells are recruited and become activated. The frequency of such naïve antigen‐specific B cells is very low in individuals who have not previously encountered the antigen [1, 2]. Notably, as complex antigens contain multiple epitopes, and B cells with different B cell receptors can recognize the same epitope with various degrees of affinity, the frequency of naïve B cells specific for any given antigen can be expected to vary, being, for most antigens, undetectable. During the primary immune response, these naïve B cells proliferate and differentiate into antibody‐secreting plasma cells (PCs) or memory B cells (B_mem_). Owing to their increased frequency, B_mem_ enable rapid secondary recall responses through their ability to undergo additional waves of proliferation—their progeny giving rise to both PCs and  B_mem_upon re‐encountering the same or related antigen [3].

Vaccination‐induced immune responses in humans and experimental animals have been extensively studied, primarily relying on antibody measurements. Murine models, however, revealed that PCs and B_mem_ arise through divergent differentiation pathways, with high‐affinity germinal center subclones being preferentially selected for PC differentiation while those of lower affinity exit lymphoid tissues as B_mem_ [4, 5, 6, 7, 8, 9, 10]. The two lineages then behave differently over time: PC survival depends on competition for limited niches in the bone marrow, whereas B_mem_ are not similarly constrained [11]. Further, the antibodies that PCs secrete have a relatively short in vivo half‐life, being about 3 weeks for IgG [12]. Serum antibodies and B_mem_ therefore constitute two distinct layers of humoral immunity [3, 4, 13]. Accordingly, antibody titers do not reflect the frequency of antigen‐specific B_mem_ [14].

Whether pre‐existing specific serum antibodies enhance or suppress the secondary B cell response has been investigated extensively, and both outcomes have been documented. Pre‐existing specific antibodies have been implicated in both immune‐stimulatory and immune‐suppressive effects (reviewed in [15, 16] and summarized in Figure S2). Stimulatory antibody feedback regulation has been attributed to antigen deposition on follicular dendritic cells [17, 18, 19, 20, 21], complement receptor 2 stimulation [15, 22, 23], and antigen uptake by dendritic cells [16, 24, 25, 26, 27]; suppressive effects have been tied to epitope masking [28, 29, 30, 31, 32, 33], B cell inhibition via the FcγR [15, 34, 35, 36], and antigen clearance [15, 16, 37].

At the level of serology, the consequence of this feedback is well documented in the literature. In the context of seasonal influenza vaccination, lower baseline hemagglutination inhibition titers are associated with larger postvaccination fold increases in both titer and antibody‐secreting cell (ASC) frequencies [38], and high pre‐existing antibody titers have been discussed to play a role in the attenuation of repeated influenza vaccine efficiency [39]. A similar inverse relationship has also been reported for COVID‐19 mRNA boosters [40, 41], with antigen clearance proposed as a causal mechanism on the basis of murine studies [41]. Furthermore, antibody feedback has also been shown to influence the epitope specificity and affinity distribution of vaccine‐induced B_mem_ [30, 42, 43], as well as limit their expansion in the case of repeated malaria vaccinations [31].

The size of the antigen‐specific B_mem_ compartment is thought to be the limiting factor predicting the efficiency of a booster immunization, as these cells are the precursors for anamnestic B cell responses. Indeed, preboost B_mem_ frequencies have been positively associated with the magnitude of the antibody response following the boost [40, 44].

However, it remains unclear to what extent the secondary B cell response is constrained by antibody feedback. If such regulatory mechanisms are prominent, the predictive value of the B_mem_ compartment at the time of the booster might be modulated by circulating antibodies. Especially in a prime‐boost setting, where specific antibodies reach high levels, it is crucial to understand how the effects exerted by the size of the preboost B_mem_ pool are balanced against the influence of pre‐existing antibodies.

To address this, we studied secondary B_mem_ expansion and its dependence on B_mem_ frequencies and specific serum antibody titers present in the same individuals before booster vaccination within a COVID‐19 mRNA prime‐boost regimen of previously immunologically naïve subjects.

For several reasons, we selected the SARS‐CoV‐2 “model” to study B_mem_ development after boosting. SARS‐CoV‐2 is unique among human viruses in that there is a defined time point at which the virus appeared and started to spread in the United States early in 2020 [45, 46, 47]. Moreover, the initial infections with SARS‐CoV‐2 were monitored in the population to a degree never before achieved for other pathogens. The individuals we tested were vaccinated soon after COVID‐19 mRNA vaccines, encoding the Wuhan‐Hu‐1 strain of SARS‐CoV‐2 Spike (S‐antigen), became widely available in 2021. Additionally, we also verified that each test subject had not been infected with SARS‐CoV‐2 before or during the observation period. In this way, we ensured that the induction of genuinely primary and secondary B cell responses in previously naïve people could be studied without pre‐existing B cell memory or SARS‐CoV‐2 infection(s) complicating the interpretation of the results.

Measuring the antibody response following vaccination is the gold standard for human immune monitoring because techniques for quantifying antigen‐specific immunoglobulins in solution are well established, and because antibodies are robust molecules after their isolation from the body [48]. However, the detection of antigen‐specific B_mem_ in mice and humans has been challenging primarily due to the very low frequency at which B_mem_ occur in the lymphocyte pool, even after clonal expansion [49], whereby in humans, frequencies of <1 in 10^5^ among peripheral blood mononuclear cells (PBMCs) are common even for B_mem_ specific for endemic viruses such as Epstein‐Barr virus, human cytomegalovirus, and seasonal influenza viruses [50].

Two techniques are commonly used for monitoring rare antigen‐specific B_mem_ in peripheral blood [49, 51]. One method entails staining B cells in PBMCs with fluorescence‐labeled antigen probes for detection by flow cytometry (FC). The second is B cell enzyme‐linked immunosorbent spot assay (ELISPOT)/FluoroSpot (here collectively designated “ImmunoSpot”), in which antigen‐specific B cells are visualized via their antibody‐derived secretory footprints and are quantified as spot‐forming units (SFUs) (Figure S1). A direct side‐by‐side comparison [51] showed that FC and ImmunoSpot both detected S‐antigen‐specific B_mem_ with comparable sensitivity and diagnostic specificity in PBMCs, provided that the B_mem_ were present in the mid‐ to high frequency range. Due to the inherent background noise of FC [49, 52] that is virtually absent in B cell ImmunoSpot assays, we hypothesized—and have since shown—that the ImmunoSpot methodology is suited for the detection of very rare antigen‐specific B_mem_ even in the <1 in 10^6^ PBMC frequency range [53]. This consideration was decisive here for a specific reason: the question we address requires the fold change of an antigen‐specific B_mem_ population to be calculated between two time points, requiring the denominator of that ratio to be measured accurately even when it is small.

## 2 Materials and Methods

### 2.1 Human Samples

Blood samples were collected internally at Cellular Technology Limited (CTL) under an Advarra‐approved IRB #Pro00043178 (CTL contract laboratory study number GL20‐16 entitled COVID‐19 Immune Response Evaluation). Blood draws were collected at three time points: prebleed (P), 14 days after the first COVID‐19 mRNA vaccination (D1), and 14 days following the second COVID‐19 mRNA booster vaccination (D2) (Figure 1A). Donors were monitored throughout the observation period for evidence of SARS‐CoV‐2 infection through measurement of nucleocapsid (NCAP)‐reactive IgG^+^ B_mem_; one individual who became NCAP‐reactive at the post‐D2 time point was excluded, leaving 20 donors in the analysis. Blood was collected into either sodium heparin or serum/SST tubes. PBMCs were isolated by standard density centrifugation and cryopreserved using serum‐free cryopreservation reagents (CTL Reagents LLC, Shaker Heights, Ohio, USA) according to previously described protocols [54]. PBMC samples were stored in vapor nitrogen tanks until testing. In line with the kinetics of the Spike‐specific plasmablast response following the initial prime‐boost COVID‐19 mRNA vaccination, scheduling of blood draws 14 days postimmunization was optimal for our study aiming to measure antigen‐specific B_mem_ following polyclonal stimulation by ELISPOT since antigen‐induced plasmablast activity is minimal at this time point [55]. For isolation of serum, blood was kept at room temperature for at least 1 h before separation by centrifugation. Serum was transferred to cryovials and subsequently stored at −20°C until testing. Details of all human donors in this manuscript, including demographics, collection dates, and vaccine manufacturer, are provided in Table S1.

**FIGURE 1:**
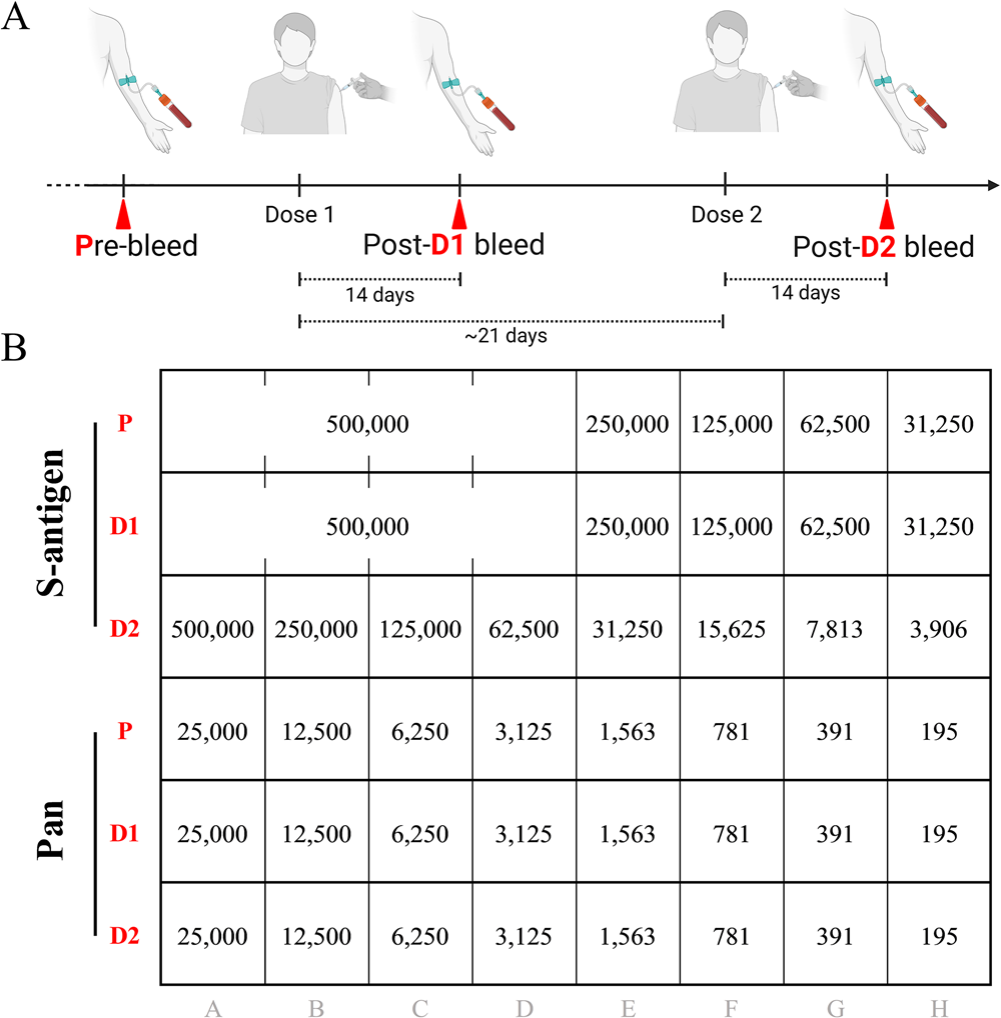
Study rationale. (A) Schematics of the vaccination regimen and blood draws. The time interval between dose 1 (D1) and dose 2 (D2) vaccinations was according to the FDA‐recommended schedule, ∼3 weeks apart. (B) ImmunoSpot plate layout permitting S‐antigen‐specific IgG^+^ B_mem_ frequency measurements over an extended range. Prevaccination (designated prebleed, P) and post‐D1 samples were seeded at 5 × 10^5^ PBMCs into four replicate wells, which permitted enumeration of individual S‐antigen‐reactive B_mem_ within a total of 2 × 10^6^ PBMCs for the detection of low‐frequency events. Additionally, a two‐fold serial cell dilution was also performed to extend the ability for precise counting when the S‐antigen‐specific secretory footprints were too crowded at the 5 × 10^5^ cells per well input for reliable enumeration. Post‐D2 samples were tested using a two‐fold serial dilution approach starting at 5 × 10^5^ PBMCs per well, yielding eight titration points. Panel (A) was created with BioRender. Hoormann, M.J. (2026) https://BioRender.com/0fbm4m6.

### 2.2 Polyclonal B Cell Stimulation

Thawing, washing, and counting of PBMCs were performed as previously described [56, 57]. Briefly, cells were transferred into polyclonal B cell stimulation cultures within 2 h of thawing after resuspension in complete medium (CM) comprising RPMI 1640 (Alkali Scientific, Fort Lauderdale, FL) supplemented with 10% fetal bovine serum (Gemini Bioproducts, West Sacramento, CA), 100 U/mL penicillin, 100 U/mL streptomycin, 2 mM L‐Glutamine, 1 mM sodium pyruvate, 8 mM HEPES (all from Life Technologies, Grand Island, NY) and 50 µM β‐mercaptoethanol (Sigma‐Aldrich, St. Louis, MO). Polyclonal stimulation of donor PBMCs was performed with Human B‐Poly‐S (CTL), containing the TLR7/8 agonist R848 and recombinant human IL‐2 [58], at 0.5–2 × 10^6^ cells/mL in 25 cm^2^ tissue culture flasks (Corning, Sigma‐Aldrich). PBMCs were cultured at 37°C, 5% CO_2_ for 5 days to promote terminal differentiation of resting B cells into ASCs before evaluation in ImmunoSpot assays as described previously [59, 60]. Based on our systematic testing [61], donor PBMC samples were prestimulated for the optimal duration of 5 days before use in ImmunoSpot assays.

### 2.3 Recombinant Proteins

Recombinant full‐length SARS‐CoV‐2 Spike (S‐antigen) protein representing the ancestral Wuhan‐Hu‐1 strain [62] was acquired from the Center for Vaccines and Immunology (CVI) (University of Georgia (UGA), Athens, GA, USA). Recombinant SARS‐CoV‐2 NCAP was purchased from the Wuhu Interferon Biological Products Industry Research Institute (Wuhu, China). Importantly, both recombinant proteins used in this study possessed a genetically encoded His affinity tag enabling their affinity coating to ImmunoSpot membrane plates.

### 2.4 Multiplexed Antigen‐Specific ImmunoSpot Assays With Affinity Capture Coating

Antigen‐specific ASCs were detected in multiplexed ImmunoSpot assays using the affinity capture coating method [63]. Low autofluorescence ImmunoSpot assay plates were preconditioned with 70% (*v/v*) EtOH (15 µL/well), followed by two washes with phosphate‐buffered saline (PBS) (180 µL/well). Assay plates were then coated with purified anti‐His antibody at 10 µg/mL in Diluent A (provided in CTL's affinity coating kits) overnight at 4°C. The following day, ImmunoSpot assay plates were washed once with 180 µL/well PBS and then coated overnight at 4°C with His‐tag‐labeled recombinant S‐antigen or NCAP at 10 µg/mL in Diluent A. Before the addition of PBMCs, ImmunoSpot assay plates were washed once with PBS, and then wells were blocked with CM for 1 h at room temperature. Immediately before plating of PBMCs, ImmunoSpot assay plates were decanted, and 100 µL prewarmed CM was added to each well.

PBMCs were harvested after 5 days of polyclonal stimulation and washed twice with PBS. PBMCs were counted using CTL's Live/Dead Cell Counting Suite on an ImmunoSpot S6 Flex Analyzer (CTL Analyzers LLC). Cell pellets were resuspended at 5 × 10^6^ live cells/mL in CM and used immediately in ImmunoSpot assays unless otherwise indicated.

To enhance the sensitivity of the S‐antigen‐specific ImmunoSpot assay for measurements of rare antigen‐reactive ASCs, prebleed (P) and post‐D1 samples were tested in four replicate wells seeded with 5 × 10^5^ polyclonally stimulated PBMCs, followed by a two‐fold serial dilution in single wells providing four additional cell titration points. Since frequencies of S‐antigen‐specific ASCs were expected to be higher following the second (booster vaccination) antigen encounter, post‐D2 PBMC samples were tested using a singlet two‐fold serial dilution approach starting at 5 × 10^5^ PBMCs per well, yielding eight titration points. Alternatively, for detection of NCAP‐reactive ASCs, PBMC samples were tested in four replicate wells seeded with 5 × 10^5^ polyclonally stimulated PBMCs; however, owing to limited cell material, some samples were only tested in two or three replicate wells. Following the addition of PBMCs, ImmunoSpot assay plates were incubated for 16 h at 37°C, 5% CO_2_. Plate‐bound SFUs, representing the secretory footprint of individual ASCs, were visualized using detection reagents included in the human IgG1/IgG2/IgG3/IgG4 Four‐Color ImmunoSpot kit (from CTL) according to the manufacturer's instructions.

### 2.5 Multiplexed Pan IgG Subclass Detection

Pan (total) IgG subclass (IgG1/IgG2/IgG3/IgG4) Four‐Color ImmunoSpot assays were performed in parallel with the antigen‐specific assays described above, using similar methods as previously described [60], to confirm ASC functionality in the polyclonally stimulated PBMC samples. Plates were coated with the anti‐κ/λ capture antibody provided in the human IgG1/IgG2/IgG3/IgG4 Four‐Color ImmunoSpot kit (from CTL). Cell suspensions were serially diluted two‐fold in singlet starting at 2.5 × 10^4^ PBMCs per well; all remaining steps were as described above.

### 2.6 ImmunoSpot Image Acquisition and SFU Counting

ImmunoSpot plates were air‐dried before scanning and counting on an ImmunoSpot Ultimate S6 Analyzer using the Fluoro‐X suite of ImmunoSpot software (Version 7.0.28) and the Basic Count mode (CTL). Individual well images were quality‐controlled as needed to remove artifacts and to improve the accuracy of counts. Only SFU counts within the linear titration range of the ImmunoSpot assay, or those from the highest cell input tested, were considered for frequency calculations and were subsequently used to extrapolate SFU counts to a fixed input of 10^6^ PBMCs.

S‐antigen‐specific B_mem_ counts at the prebleed time point were too low to establish reliable frequencies. To enable plotting them on a logarithmic scale in the corresponding figure, we assigned them a value of 0.5 SFU/10^6^ PBMCs. Notably, since only IgG1^+^ and IgG3^+^ SFUs were detected in S‐antigen‐specific ImmunoSpot assays, the resulting counts were combined and are denoted as IgG.

### 2.7 ELISA

S‐antigen‐specific IgG levels in serum samples were quantified according to previously described methods with minor modifications [14]. In brief, microtiter enzyme‐linked immunosorbent assay (ELISA) plates (Immulon 4HBX, flat‐bottom, Thermo Fisher Scientific, Waltham, MA, USA) were coated with 80 µL/well of S‐antigen at 2 µg/mL in carbonate buffer at pH 9.3 overnight at 4°C. Plates were then decanted and blocked with ELISA blocking buffer consisting of PBS supplemented with 2% (w/v) bovine serum albumin (Sigma‐Aldrich, St. Louis, MO, USA) and 0.1% (v/v) Tween 20 for 1.5 h at room temperature. After blocking, serially diluted serum samples were incubated in the microtiter plates overnight at 4°C. The following day, microtiter plates were washed with PBS before the addition of horseradish peroxidase‐conjugated anti‐human IgG detection reagent (from CTL) and incubated for 1.5 h at room temperature. After washing the microtiter plates with PBS, 100 µL/well of 1‐Step TMB ELISA Substrate Solution (Thermo Fisher Scientific) was added to develop the assay. Conversion of the TMB substrate was terminated by addition of 100 µL/well of 2 M HCl, and the optical density was measured at 450 nm using a SpectraMax 190 plate reader (Molecular Devices, San Jose, CA, USA). The abundance of S‐antigen‐specific IgG in the serum samples was determined by interpolating OD_450_ values into µg/mL of IgG equivalents using SpotStat (Version 1.6.6.0, CTL). The underlying standard curves were generated by directly coating decreasing quantities of a reference IgG preparation (from Athens Research and Technology, Athens, GA, USA) in duplicates into designated wells of each assay plate.

### 2.8 Statistical Methods

Statistical analyses were performed in GraphPad Prism 10 (Version 10.6.1; San Diego, CA, USA) unless otherwise stated. Differences between the post‐D1 and post‐D2 time points were assessed by the Wilcoxon matched‐pairs signed‐rank test, and Spearman rank correlation was used for all association analyses shown in Figures 4 and 5. Fold changes in S‐antigen‐specific ASC frequencies were additionally compared between donors with post‐D1 serum IgG levels below versus above the cohort median (∼30 µg/mL equivalents) by a Mann–Whitney test. Per‐donor differences between prebleed and post‐D1 SFU counts were assessed by an exact Poisson test (Microsoft Excel; Table S2). Linearity of the ImmunoSpot cell titration (Figure 2C) was assessed by linear regression. All tests were two‐tailed, and *p* < 0.05 was considered significant; *r*_S_ and *p* values are shown in the respective figures.

**FIGURE 2:**
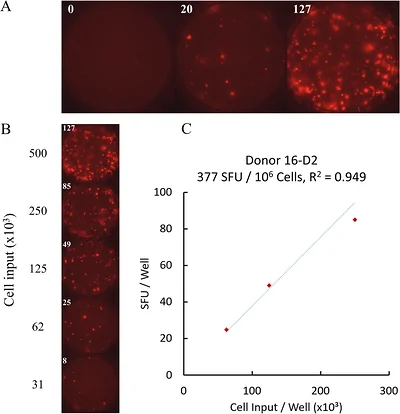
ImmunoSpot data from a representative subject (Donor 16). (A) Wells from the same donor containing either the prevaccination (P) (the well on the left), post‐D1 (the middle well), or post‐D2 (well on the right) samples at 5 × 10^5^ PBMCs per well. (B) Two‐fold serial dilution of the post‐D2 bleed sample. The PBMCs were seeded in the numbers specified, according to the plate layout shown in Figure 1B. Secretory footprints generated by S‐antigen‐specific B_mem_‐derived IgG^+^ ASCs were visualized as described in Section 2. When the secretory footprints (SFUs) were too crowded at 5 × 10^5^ PBMCs/well for accurate enumeration, the frequencies were calculated from the two‐fold dilution series as shown in panel (C), and as described in Section 2. Data shown in panels (A) and (B) were generated in a single experiment in which the donor's PBMCs were tested in parallel under identical experimental conditions.

## 3 Results and Discussion

### 3.1 Overall Rationale

The timeline of sample collection in our study and the plate layout of the SARS‐CoV‐2 Spike (S‐antigen)‐specific ImmunoSpot assays that were performed are depicted in Figure 1. In essence, we focused on determining S‐antigen‐specific B_mem_ frequencies and antibody titers before (prebleed, P), 14 days after the first dose (post‐D1), and 14 days after the second dose (post‐D2) of the COVID‐19 mRNA vaccine (Comirnaty) encoding the S‐antigen of the prototype SARS‐CoV‐2 (Wuhan‐Hu‐1 strain) virus. The timing of the booster administrations was based on WHO recommendations [64].

B cell reactivity to the SARS‐CoV‐2 NCAP, which was not included in the vaccine, was also measured to control for the possibility that infection with SARS‐CoV‐2 occurred before, or during, the observation period. One individual did indeed become positive for NCAP‐specific B_mem_‐derived IgG^+^ ASC reactivity at the post‐D2 time point and was therefore excluded from the analysis.

The overall goal of this study was to determine whether secondary clonal expansions of B_mem_ were preprogrammed when the antigen is re‐encountered or were rather defined by feedback regulation. If preprogrammed, one would expect that individuals exhibiting higher frequencies of B_mem_ after the primary vaccination and therefore possessing increased numbers of precursor cells available for the secondary antigen encounter, would develop higher numbers of B_mem_ levels following renewed clonal expansion. In contrast, if antibody feedback inhibition is the dominating factor limiting secondary clonal expansion of antigen‐specific B_mem_, we would not expect a strong association between post‐D1 and post‐D2 B_mem_ frequencies but rather an inverse relationship of post‐D1 antibody levels and the subsequent B_mem_ expansion.

### 3.2 Divergence of B_mem_ Frequencies and Serum Antibody Titers

Antigen‐specific B_mem_ can occur over a very wide frequency range in PBMCs [50, 65]. The plate layout we used for ImmunoSpot testing accommodated such differences (Figure 1B). The PBMCs of each donor were plated at 5 × 10^5^ cells/well into four replicate wells, followed by additional two‐fold serial dilutions of the sample, tested as singlets. This layout enables the detection of even one S‐antigen‐specific B_mem_‐derived IgG^+^ ASC in 2 × 10^6^ PBMCs, although Poisson noise limits the accuracy of frequency estimates in this range, while the serial dilution permits the precise enumeration of B_mem_ occurring at higher frequencies [53]. From the linear range of SFU counts, the frequency of antigen‐specific B_mem_‐derived IgG^+^ ASCs can be extrapolated from single‐well serial dilutions with nearly identical precision as tests employing replicate wells for each cell dilution [50]. In this manner, ImmunoSpot assays were performed to establish the frequency of S‐antigen‐specific B_mem_‐derived ASCs secreting each of the four IgG subclasses. Additionally, in parallel, we also evaluated the PBMC samples in pan (total) IgG ImmunoSpot assays to determine the frequency of ASCs capable of secreting the different IgG subclasses irrespective of their antigen specificity.

Original well images depicting the testing of a representative subject (Donor 16) for S‐antigen‐specific B_mem_‐derived IgG1^+^ ASCs at the three PBMC collection time points are shown in Figure 2A,B, while the results for all donors are summarized in Figure 3A. Notably, owing to the elevated frequency of S‐antigen‐specific IgG^+^ B_mem_ detected at the post‐D2 time point, serial dilution of the Donor 16‐D2 sample permitted an accurate frequency measurement (Figure 2C). Moreover, at the prebleed time point, too few S‐antigen‐reactive IgG^+^ B_mem_ were detected among the 2 × 10^6^ PBMCs tested to establish reliable frequencies. This observation is consistent with the notion that such donors were essentially naïve to the SARS‐CoV‐2 S‐antigen before their first vaccination [53] and that cross‐reactively‐primed IgG^+^ B_mem_ [66] in our cohort occurred at very low, not quantifiable frequencies. As S‐antigen‐specific B_mem_ frequencies at the prebleed time point were below the limit of reliable quantification, all prebleed values were plotted at 0.5 SFU/10^6^ PBMCs to permit display on a logarithmic scale in Figure 3A; the underlying raw counts are provided in Table S2.

**FIGURE 3:**
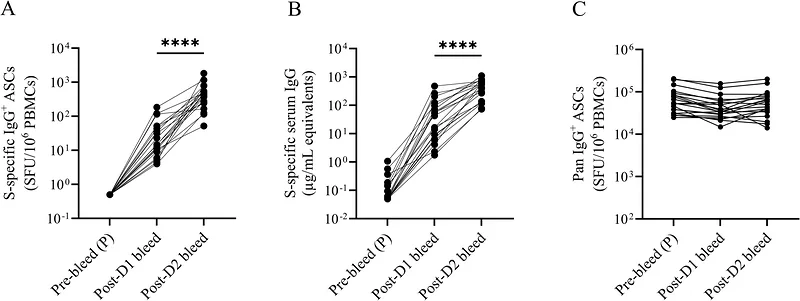
The S‐antigen‐specific B cell response at the cohort level. (A, B) S‐antigen‐specific IgG^+^ B_mem_ frequencies (panel A) and S‐antigen‐specific serum IgG concentrations (panel B) were measured in the individual donors (each represented by a dot) at the prebleed (P), post‐D1, and post‐D2 time points, as specified. As S‐antigen‐specific B_mem_ frequencies at the prebleed time point were below the limit of reliable quantification, all prebleed values were plotted at 0.5 SFU/10^6^ PBMCs to enable visualization on a logarithmic scale; the underlying raw counts are provided in Table S2. Likewise, serum IgG antibody levels below our limit of quantification were assigned a value of 0.05 µg/mL for graphing purposes. (C) The frequencies of pan (total) IgG^+^ ASCs for the three time points. The ImmunoSpot assays for testing the PBMCs, and ELISA to test the sera were performed as described in Section 2. In panels (A) and (C), the frequencies of S‐antigen‐specific and pan IgG^+^ ASCs were measured in a single experiment in which all PBMC samples were tested in parallel under identical experimental conditions. In panel (B), S‐antigen‐specific serum IgG levels were measured in two independent experiments, and results were combined. Statistical significance between post‐D1 and post‐D2 time points was determined using Wilcoxon matched‐pairs signed‐rank tests. ****p < 0.0001.

S‐antigen‐specific IgG^+^ B_mem_ became detectable in all subjects by day 14 post‐D1. As expected, considerable interindividual B_mem_ frequency differences were seen in this cohort, ranging from 4 to 184 IgG^+^ ASCs/10^6^ PBMCs (Figure 3A). While the responses after D1 were low in some subjects, the induction of B_mem_ was evident even in these individuals and statistically significant in each case (exact Poisson test; Table S2). These data suggest that the magnitudes of initial clonal expansions differed substantially between individuals. To this end, quantification of S‐antigen‐specific serum IgG levels also showed a relatively high degree of heterogeneity (Figure 3B), as typically observed for postvaccination antibody responses [67, 68, 69, 70, 71, 72, 73].

Importantly, reliable B_mem_ measurements by ImmunoSpot require that PBMC samples are cryopreserved and thawed such that the cells maintain full functionality [50]. As shown in Figure 3C, the overall functionality of the PBMCs (i.e., number of pan IgG^+^ ASCs detected following polyclonal stimulation) was similar at the prebleed, post‐D1, and post‐D2 time points. Therefore, the observed differences in the frequency of S‐antigen‐specific B_mem_ reactivity were inherent to each donor's B_mem_ composition at the time of blood collection. To further substantiate this point, Figure S3 demonstrates that no significant relationship could be found between the antigen‐specific and pan IgG^+^ ASC response magnitudes within donors.

While Figure 3A,B displays S‐antigen‐specific B_mem_ frequencies and serum IgG levels, respectively, the correlation between those variables is shown in Figure 4. When comparing the relationship between B_mem_ frequencies and IgG titers at the post‐D1 time point, a correlation was clearly observable (Figure 4A); notably, the correlation fails to reach statistical significance according to Spearman correlation analysis when very low responding donors are excluded. Following the second vaccination, this relationship was lost (Figure 4B), indicating that the humoral arm of the response (serum IgG, reflecting PC activity) and its cellular arm (generation of B_mem_) diverge as the response matures. Serum antibody levels and B_mem_ therefore dissociate over the course of the response and are not reflective of each other. But do increased frequencies of B_mem_ predispose to stronger secondary B cell responses, and if not, is the reactivity regulated by antibody feedback?

**FIGURE 4:**
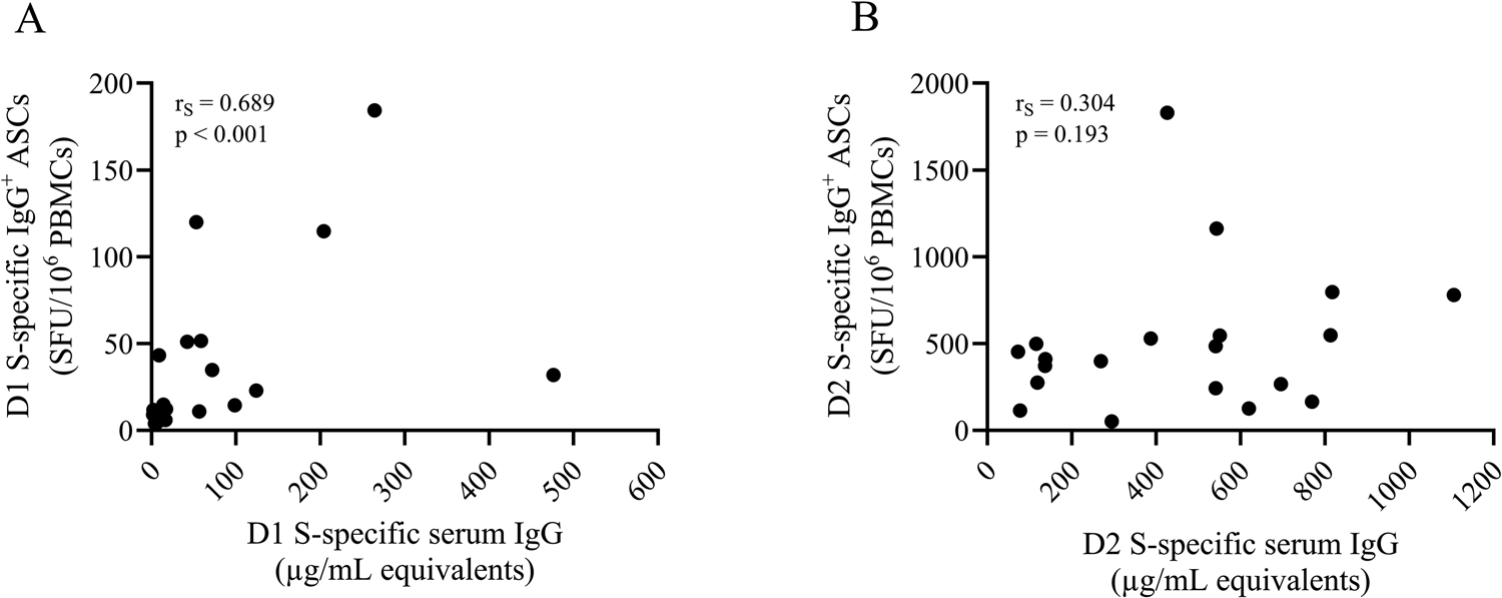
Correlation analyses of S‐antigen‐specific serum IgG levels and B_mem_ frequencies in post‐D1 (A) and post‐D2 (B) bleeds. The results shown in Figure 3 for these two time points are broken down here for the individuals, with each dot representing one of the vaccinees. Spearman correlation analyses were performed. r_S_ and *p*‐values are denoted in the corresponding graphs.

### 3.3 The Magnitude of the Secondary B Cell Response Is Constrained by Preboost Serum IgG Levels Rather Than Determined by Preboost B_mem_ Frequencies

Having measured both arms (humoral and cellular) of the B cell response at the post‐D1 and post‐D2 time points, we next sought to assess how they were correlated. Figure 5A shows that higher numbers of S‐antigen‐specific IgG^+^ B_mem_ before administering the booster vaccination (D1 S‐antigen‐specific IgG^+^ ASCs, on the *x*‐axis) did not predispose to a higher frequency of such B_mem_ at the post‐D2 time point (D2 S‐antigen‐specific IgG^+^ ASCs, on the *y*‐axis). Therefore, the presence of a larger antigen‐specific B_mem_ pool present at administration of the booster vaccination did not imply a greater degree of recruitment and engagement of B_mem_ into a proportionally larger secondary B cell expansion. Similarly, starting with a greater number of antigen‐specific B_mem_ before boosting (Figure 5B, *x*‐axis) did not lead to a predictably stronger antigen‐specific antibody response following the booster vaccination (Figure 5B, *y*‐axis). Increased antigen‐specific serum IgG levels before the boost vaccination (Figure 5C, *x*‐axis) were positively correlated with a higher antigen‐specific antibody response thereafter (Figure 5C, *y*‐axis), according to Spearman correlation. Notably, however, donors who displayed relatively low levels of S‐antigen‐specific serum IgG preboost achieved the highest levels of such after the second vaccination.

**FIGURE 5:**
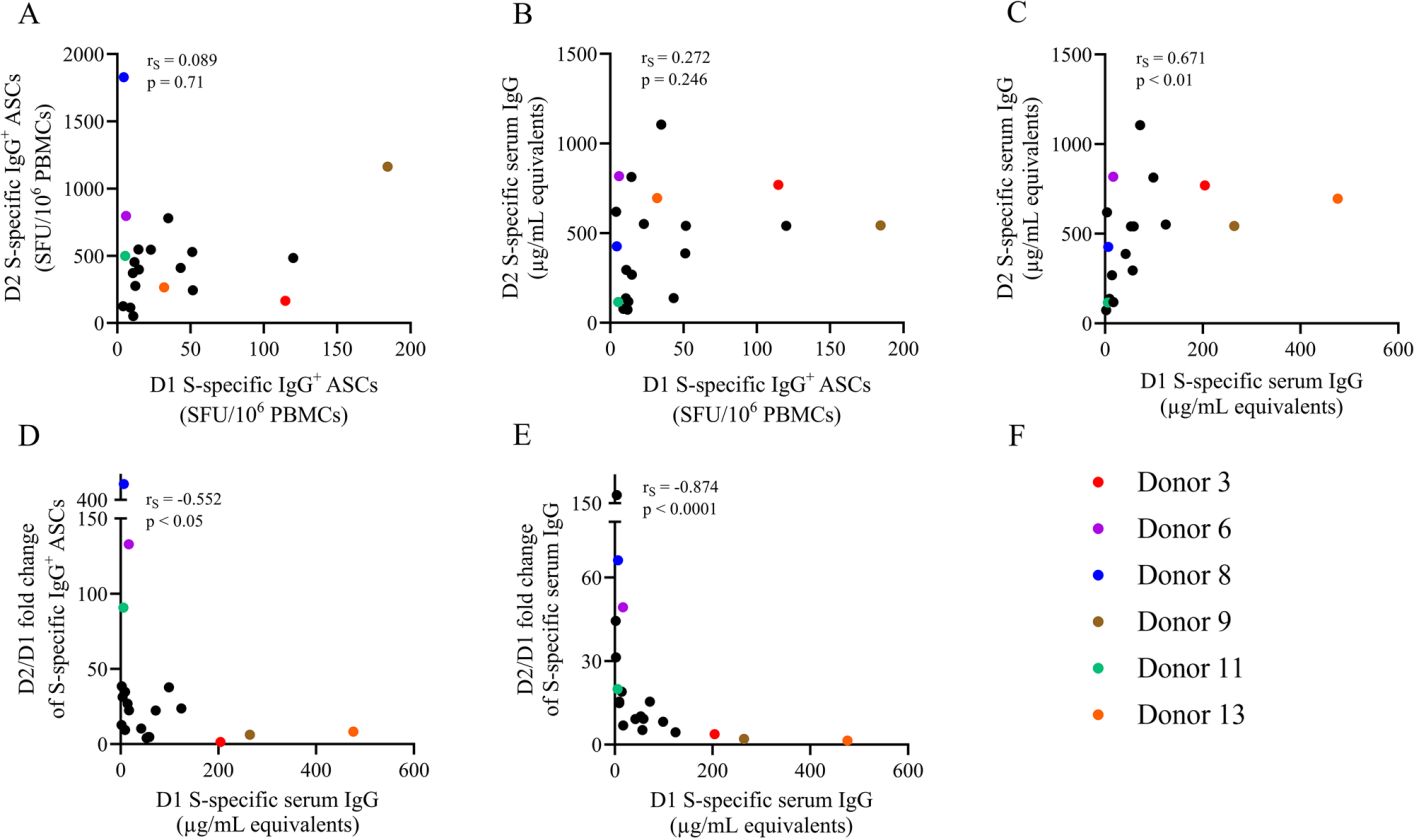
The secondary B cell response is not predetermined by pre‐existing antigen‐specific B_mem_ frequencies, and pre‐existing specific antibodies are associated with reduced secondary expansion of specific B cells. The data shown in Figure 3 are represented here for the individual donors, each as a dot, as follows: (A) post‐D1 S‐antigen‐specific B_mem_ frequencies on the *x*‐axis vs. post‐D2 S‐antigen‐specific B_mem_ frequencies on the *y*‐axis. (B) Post‐D1 S‐antigen‐specific B_mem_ frequencies on the *x*‐axis vs. post‐D2 S‐antigen‐specific serum IgG levels on the *y*‐axis. (C) Post‐D1 S‐antigen‐specific serum IgG levels on the *x*‐axis vs. post‐D2 S‐antigen‐specific serum IgG levels on the *y*‐axis. (D) Post‐D1 S‐antigen‐specific serum IgG levels on the *x*‐axis vs. the expansion of B_mem_ from post‐D1 to post‐D2 bleeds on the *y*‐axis. (E) Post‐D1 S‐antigen‐specific serum IgG levels on the *x*‐axis vs. increase in S‐antigen‐specific serum IgG from post‐D1 to post‐D2 on the *y*‐axis. (F) The identities of six donors of note are depicted by the respective color‐coding scheme to enable their tracking in the respective panels. Spearman correlation analyses were performed. *r*_S_ and *p*‐values are denoted in the corresponding graphs.

Collectively, such data suggest that the secondary engagement of antigen‐specific precursor cells (i.e., the B_mem_) is not solely driven by the re‐exposure to the antigen, but instead is governed by additional regulatory control mechanisms. Indeed, in our donor cohort both the fold increase (clonal expansion) of S‐antigen‐specific IgG^+^ B_mem_ and the resulting boost in serum IgG levels (in vivo differentiation into ASCs) were inversely correlated with the S‐antigen‐specific antibody levels measured at the post‐D1 time point (D1 S‐antigen‐specific serum IgG on the x‐axes of Figure 5D,E versus the fold‐increase of S‐antigen‐specific IgG^+^ ASCs on the y‐axis of Figure 5D, and the fold‐increase of S‐antigen‐specific serum IgG on the y‐axis of Figure 5E). In both cases, the inverse association was statistically significant, as determined by Spearman correlation analysis. Additionally, Figure S4 directly compares the fold‐increase of antigen‐specific B_mem_ between donors with low versus high post‐D1 S‐antigen‐specific serum IgG, demonstrating a significantly greater expansion in the low S‐antigen‐specific serum IgG group.

## 4 Concluding Remarks

Adaptive immune responses are based on antigen‐driven expansion of specific clones, as originally predicted by Burnet's clonal selection theory [74]. The rationale of vaccinations, including booster administration of the antigen, has been based on this fundamental concept. While correct beyond doubt for primary responses, the assumption has been that the B cell system will stereotypically respond with renewed clonal expansions to each renewed antigen encounter. For the naïve state, when there are by definition no specific antibodies present to exert feedback inhibition, primary clonal expansions allow antigen‐specific naïve B cells to clonally expand to reach frequencies that become detectable (see Table S2; Figure 3A). It is conceivable that the development of the primary S‐antigen‐specific antibody response within the first week following the initial COVID‐19 mRNA vaccination [75] can already restrain further clonal expansion of antigen‐specific B cells [32]. Previously primed, cross‐reactive B_mem_ and antibodies may also exert an effect on the magnitude of the primary B cell response.

Overall, while higher antibody levels and B_mem_ frequencies were present after the booster vaccination, the increase is relatively minor (maximally 400‐fold, on average 45‐fold, corresponding to maximally nine, on average five cell divisions, respectively, Figures 3 and 5) relative to the primary B cell response, which starts from a naïve precursor frequency, orders of magnitude lower. Importantly, in every subject of our cohort, the booster vaccination had a positive effect on B_mem_ and antibody generation.

The finding that pre‐existing antibody levels are inversely related to the magnitude of the subsequent antibody response is not novel [38, 39, 40, 41]. Our serological data (Figure 5E) reproduce this relationship within a two‐dose prime‐boost schedule. What the present study adds is a measurement of the B_mem_ compartment alongside it, at both the preboost and postboost time points, in subjects whose immune history with respect to the immunogen is fully defined. Two observations follow. First, the frequency of S‐antigen‐specific IgG^+^ B_mem_ present at the time of boosting did not predict the frequency attained after the boost, nor the antibody level attained after the boost (Figure 5A,B). Second, the preboost S‐antigen‐specific serum IgG level was inversely associated with the expansion of the B_mem_ compartment itself (Figure 5D).

Consequently, our data suggest that the B cell system does not stereotypically respond to antigen re‐encounter with the initiation of a robust secondary B cell response, but does so only when antigen‐specific antibodies are not present at sufficiently high levels to be inhibitory. Therefore, in our study, the level of pre‐existing specific antibody was a better predictor of the secondary response than the size of the B_mem_ compartment available to mount it.

Activation and clonal expansion of B_mem_ induced by the re‐encounter of the antigen and its inhibition by specific antibodies are concurrent events regulating the net outcome of the anamnestic immune response that ensues. In the prime‐boost situation, antibody levels induced by the primary antigen encounter (D1) can reach high levels. Our data show that in individuals with particularly high antibody levels, B_mem_ expansion does not reach the level that the preboost B_mem_ frequencies would predict. In contrast, it has been reported that in individuals who have been given a third vaccine dose 9 months after the initial prime‐boost vaccination, by which time serum antibody levels have declined, the preboost B_mem_ frequencies were positive predictors of the subsequent antibody responses [40].

In light of these findings, we contend that it is warranted to revisit vaccination regimes stipulating the administration of boosters at fixed intervals, at least where specific antibody elicited by the preceding dose has not yet waned, as this assumes that the immune system reacts stereotypically with renewed rounds of clonal expansion and subsequent B_mem_ differentiation following a given antigen stimulus. Indeed, clinical evidence for vaccine attenuation is emerging [76, 77]. By demonstrating that the restrictive force of pre‐existing antibodies outweighs the predictive value of the preboost B_mem_ pool, our study highlights the critical need to account for such regulatory mechanisms when optimizing booster schedules. Extending the immune monitoring data on which clinical vaccination protocols are based by routinely incorporating cellular measurements is likely to unveil novel insights and guide future vaccination strategies aiming at eliciting durable and protective immunity.

## 5 Limitation of our Study

Our study was limited in scope since we only evaluated paired PBMC samples originating from 20 donors. While our cohort included subjects of various ages and both males and females, it lacked racial diversity since only one non‐Caucasian participant was included. Additionally, the post‐D1 blood draws occurred 2 weeks after the initial vaccination and ∼1 week in advance of the booster vaccination. Therefore, it is plausible that frequencies of S‐antigen‐specific IgG^+^ B_mem_ and/or serum IgG levels may have continued to increase (or decrease) slightly during this week. Nevertheless, given the differences in response magnitudes observed between individual test subjects in our cohort, we consider it highly unlikely that fundamental changes in their response hierarchies would have occurred before the administration of their second COVID‐19 mRNA vaccine. Likewise, our donor cohort was not profiled for S‐antigen‐specific serum IgG levels or IgG^+^ B_mem_ frequencies beyond the post‐D2 time point. Consequently, we were unable to assess the prognostic relationship of serum IgG and B_mem_ frequencies measured acutely after the COVID‐19 mRNA vaccine regimen with levels achieved/maintained at longer‐term time points. Also, in contrast to conventional protein subunit/split inactivated vaccines in which the amount of antigen can be measured before inoculation, another variable in our donor cohort that could influence the magnitude of the ensuing B cell response was the tissue restriction and duration of S‐antigen expression following the COVID‐19 mRNA vaccinations [75, 78, 79]. Finally, our study was not designed to identify the mechanism of the suppression. As the association we report is with total S‐antigen‐specific serum IgG, epitope masking, FcγRIIB‐mediated inhibition, and antigen clearance all remain compatible with our data, since each scales with the concentration of specific antibody.

## Author Contributions

All authors fulfilled the ICMJE recommended criteria for authorship, with their major contribution being as follows: Conceptualization: M.J.H., P.V.L., and G.A.K.; Methodology: M.J.H., N.B., L.Y., and G.A.K.; Formal analysis: M.J.H. and G.A.K.; Investigation: M.J.H., L.Y., N.B., and G.A.K.; Resources: A.V.G. and M.T.L.; Data curation: M.J.H., A.V.G., M.T.L., and G.A.K.; Writing – original draft preparation: M.J.H. and P.V.L.; Writing – review and editing: M.J.H., S.K., M.T.L., G.A.K., and P.V.L.; Supervision: P.V.L.; Project administration: G.A.K. This publication serves as part of M.J.H.’s doctoral thesis to be submitted to the University of Bonn, Germany. All authors have read and agreed to the published version of the manuscript.

## Funding

This research was funded by the R&D budget of Cellular Technology Limited (CTL). S.K. is a member of the Excellence Cluster Immunosensation^3^ funded by the DFG under Germany's Excellence Strategy ‐ EXC2151‐390873048.

## Institutional Review Board Statement

PBMCs from COVID‐19 mRNA‐vaccinated donors were collected internally at CTL under an Advarra‐Approved IRB #Pro00043178 (CTL study number: GL20‐16 entitled COVID‐19 Immune Response Evaluation).

## Informed Consent Statement

Informed consent was obtained from all individuals sampled internally under an Advarra‐Approved IRB #Pro00043178 (CTL study number: GL20‐16 entitled COVID‐19 Immune Response Evaluation).

## Conflicts of Interest

P.V.L. is founder, president, and CEO of Cellular Technology Limited (CTL), a company that specializes in immune monitoring by ImmunoSpot. M.T.L. is a co‐founder and CSO of CTL. A.V.G. is a current employee of CTL. L.Y., N.B., and G.A.K. are former employees of CTL. M.J.H. and S.K. declare no conflicts of interest. This study was funded by CTL, and the funder directed the study design, collection, analysis, interpretation of data, and the writing of this article, and made the decision to submit it for publication.

## Supporting information



Figure S1: Illustration of antigen‐specific and pan (total) B cell ImmunoSpot test principles; Figure S2: Overview of antibody feedback mechanisms; Figure S3: Lack of correlation between S‐antigen‐specific and pan IgG^+^ ASC frequencies.; Figure S4: Expansion of S‐antigen‐specific B cells in donors with higher or lower post‐D1 S‐antigen‐specific serum IgG levels. Table S1: Details of donor cohort. Table S2: Raw SFU counts from replicate wells at the prebleed and post‐D1 time points. **Supporting File**: eji70315‐sup‐0001‐SuppMat.pdf.

## Data Availability

The data generated in this study will be made available by the authors, without undue reservation, to any qualified researcher.
