# Supplementary material for: The Magnitude of the Secondary B Cell Response is Shaped by Pre‐Existing Antibodies Rather Than the Number of Memory B Cells Present
Source: Eur J Immunol. 2026 Nov 11;56(11):e70315. doi: 10.1002/eji.70315 (PMC13755499; doi:10.1002/eji.70315)
Supplement: Supplementary file 1 [file EJI-56-e70315-s001.pdf]

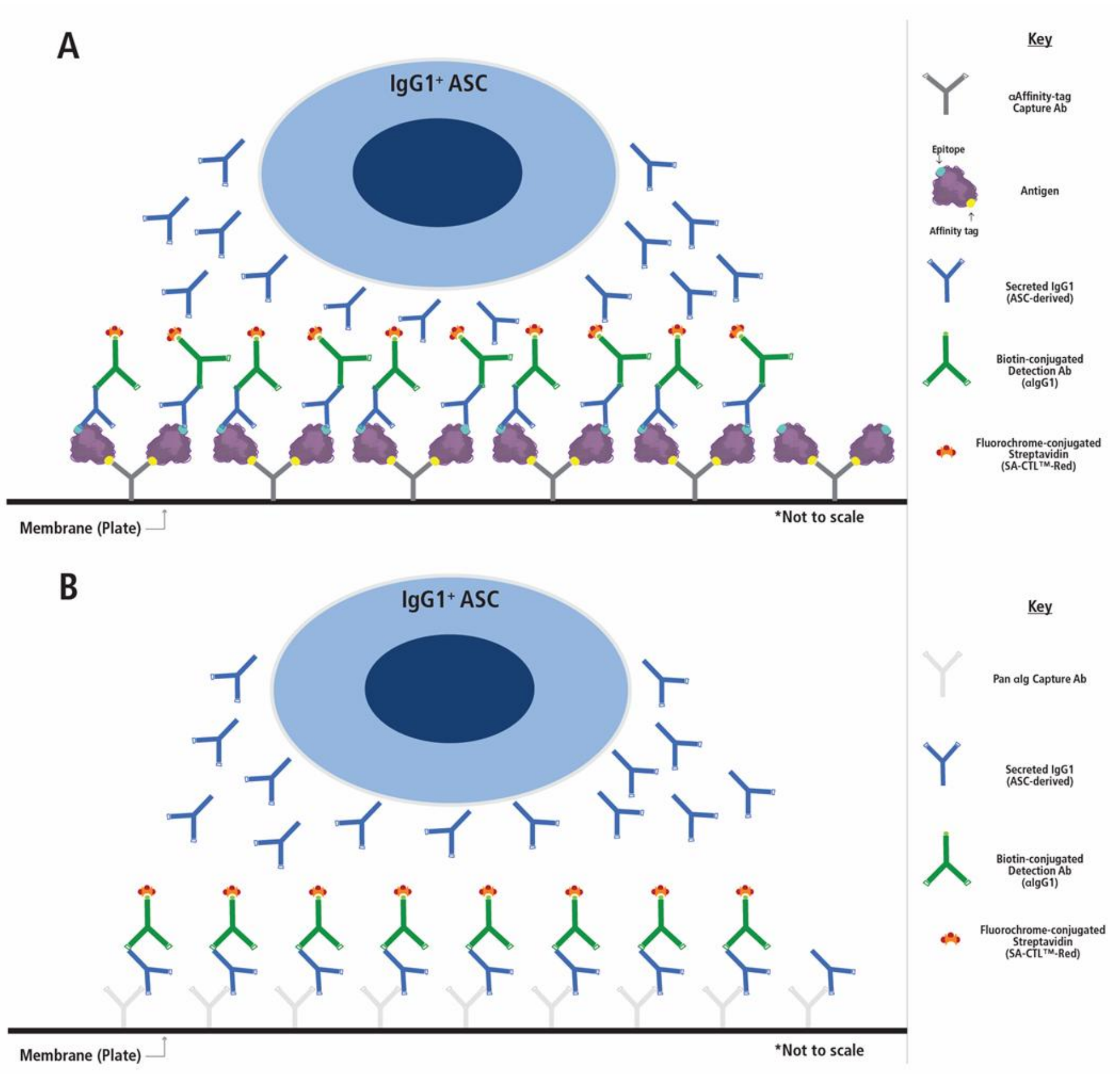

Supplementary Figure S1. Illustration of antigen-specific and pan (total) B cell ImmunoSpot® test principles. A) In an antigen-specific detection assay the membrane can be coated directly (not shown) or via affinity capture (as shown) with the antigen of interest to achieve a high density of antigen coating and maximal detection of antigen-specific antibody-secreting cells (ASCs) [63]. When ASCs are plated onto such an antigen-coated surface, the antibodies produced by an antigen-specific ASC (shown in blue), as opposed to antibodies produced by ASCs specific for other antigens, will be retained on the membrane in close proximity to where the ASC resided on the membrane as a secretory footprint. B) Alternatively, in a pan (total) IgG detection assay the Ig produced by ASCs is captured irrespective of its antigen specificity by an anti-species antibody coated onto the membrane (e.g. goat anti-human Igκ/λ) in close proximity to where the ASC resided on the membrane instead of being secreted into the culture supernatant. In both (A) antigen-specific or (B) pan IgG1 ImmunoSpot® assays, plate-bound IgG1-derived secretory footprints are visualized using an anti-human IgG1-specific detection antibody (depicted in green), followed either by deposition of a precipitating visible substrate in an ELISPOT (not shown) or through selective excitation of the fluorophore associated with the detection antibody and visualization using a suitable instrument in a FluoroSpot (as shown).

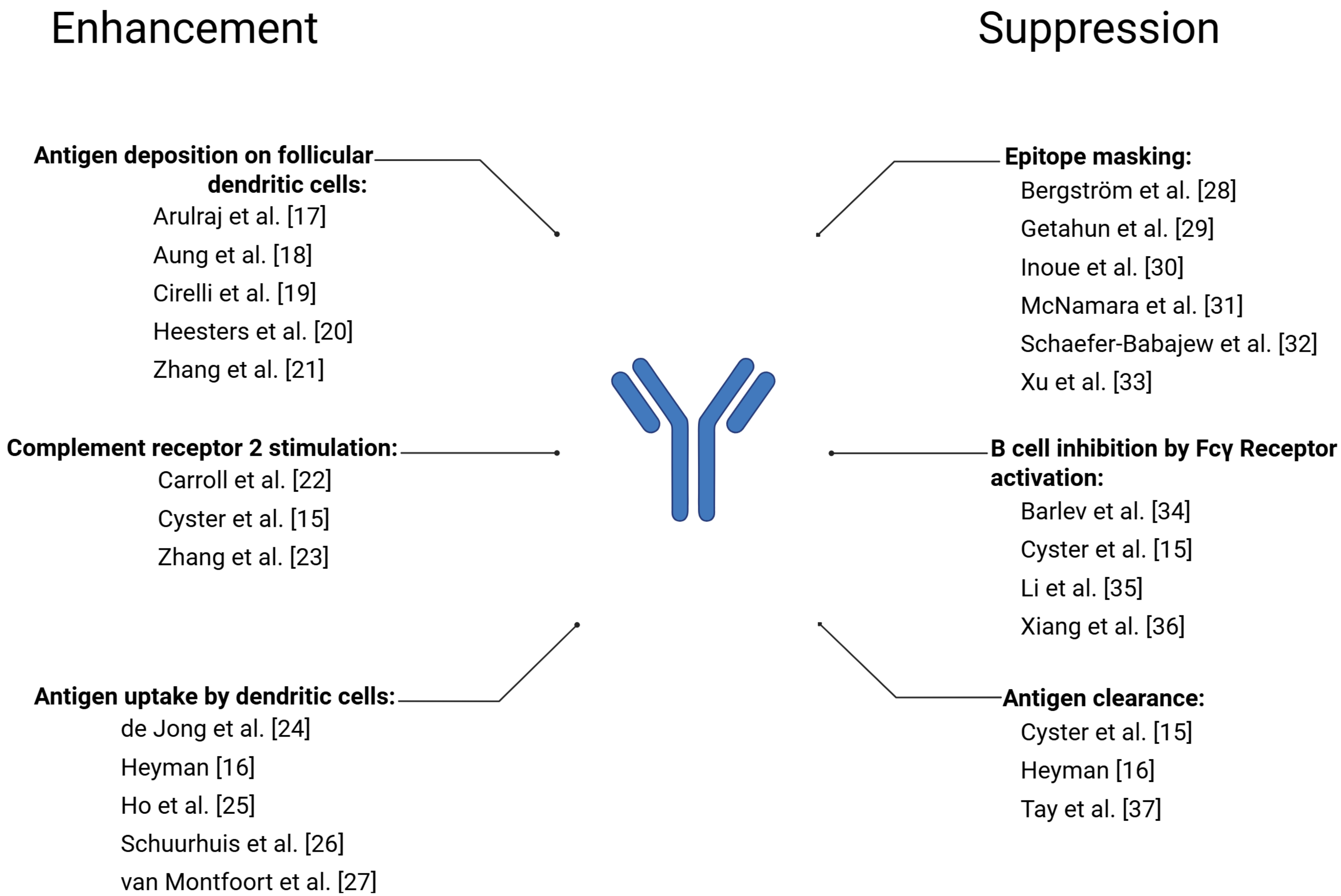

Supplementary Figure S2. Overview of antibody feedback mechanisms. References detailing each mechanism are denoted and numbering of references corresponds to the main text (illustration of antibody originates from BioRender).

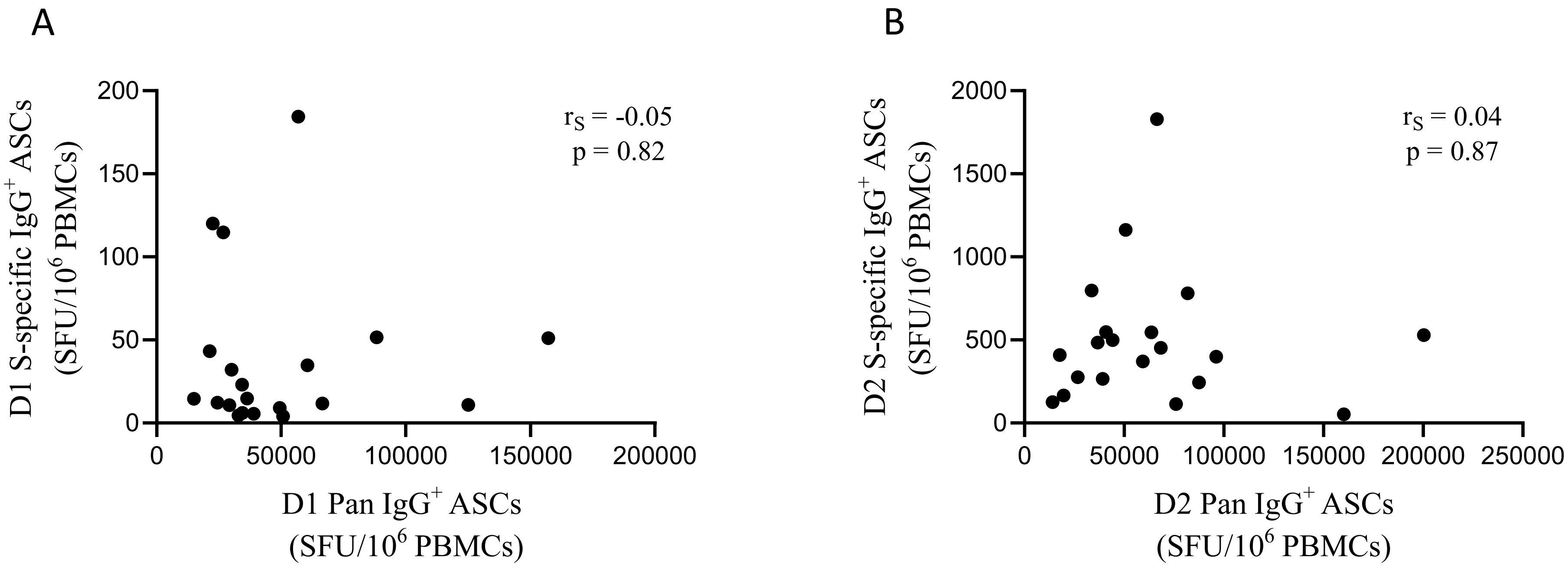

Supplementary Figure S3. Lack of correlation between S-antigen-specific and Pan IgG⁺ ASC frequencies. For each vaccinee, frequencies of S-antigen-specific IgG⁺ ASCs and Pan IgG⁺ ASCs are shown, with panel A representing the post-D1 and panel B the post-D2 timepoint. Spearman rank correlation analyses were performed and the resulting r_S and p values are denoted.

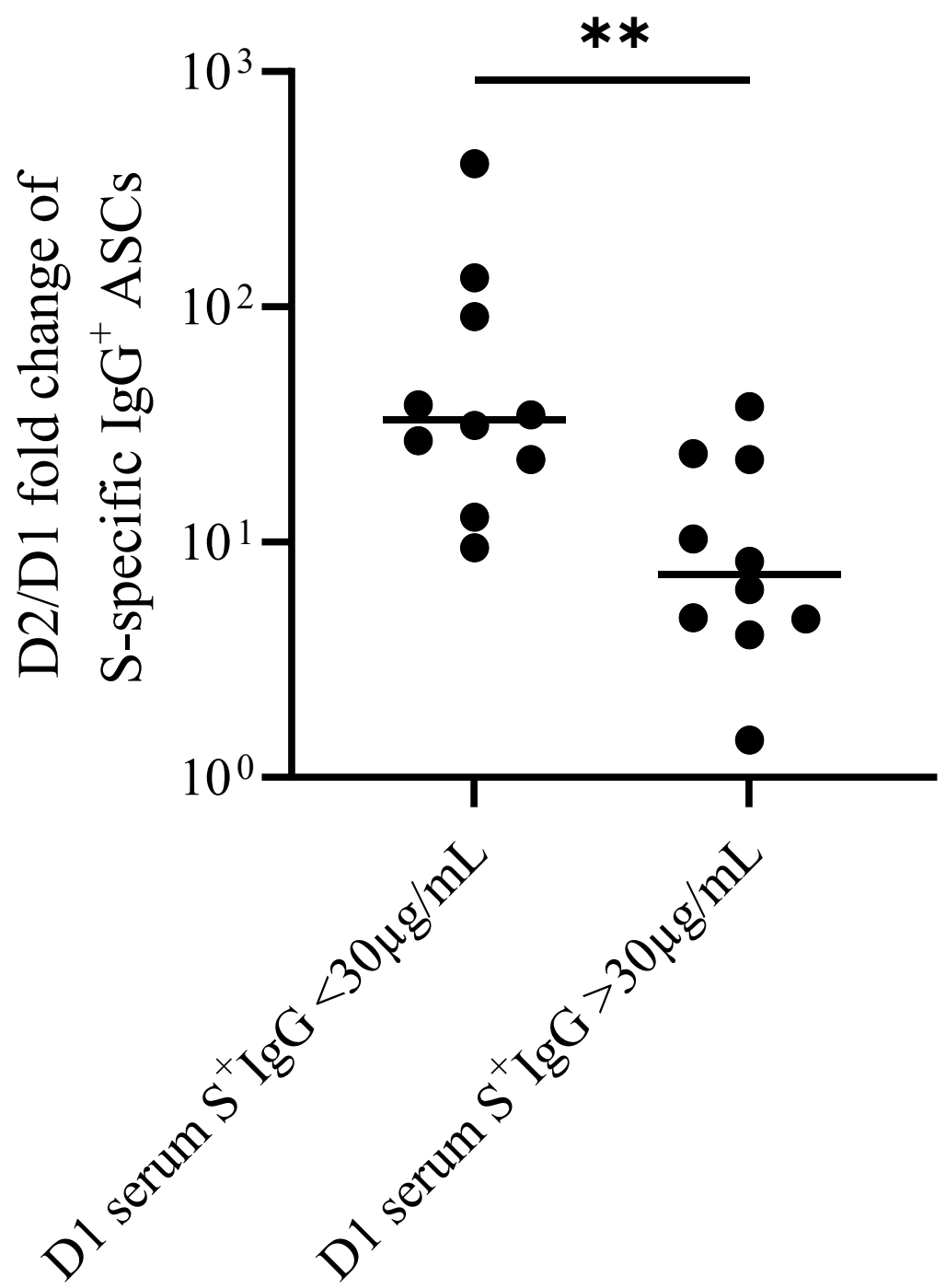

Supplementary Figure S4. Expansion of S-antigen-specific B cells in donors with higher or lower post-D1 S-antigen-specific serum IgG levels. Fold change in S-antigen-specific IgG^+ ASC frequencies between the post-D1 and post-D2 timepoints is shown for vaccinees, stratified into two groups based on the cohort median of post-D1 S-antigen-specific serum IgG levels (below or above ~30 μg/mL equivalents). Differences between the two groups were determined using a Mann-Whitney test; ** $p < 0.01$. Horizontal lines indicate the group medians.

Donor ID	Age (at Pre-bleed)	Race	Gender	Vaccine type	Pre-bleed sample collection date	Post-D1 bleed sample collection date (14 days after first vaccination)	Post-D2 bleed sample collection date (14 days after second vaccination)
Donor 1	55-59	White	M	Pfizer/BioNTech	4.5.2021	4.23.2021	5.14.2021
Donor 2	35-39	White	M	Pfizer/BioNTech	4.2.2021	4.19.2021	5.10.2021
Donor 3	18-19	White	M	Pfizer/BioNTech	4.28.2021	5.17.2021	6.7.2021
Donor 4	18-19	White	M	Pfizer/BioNTech	3.29.2021	4.19.2021	5.17.2021
Donor 5	60-65	White	F	Pfizer/BioNTech	3.15.2021	4.5.2021	4.26.2021
Donor 6	30-34	White	F	Pfizer/BioNTech	4.1.2021	4.21.2021	5.11.2021
Donor 7	30-34	White	F	Pfizer/BioNTech	3.30.2021	4.16.2021	5.4.2021
Donor 8	50-54	White	F	Pfizer/BioNTech	3.15.2021	4.5.2021	4.26.2021
Donor 9	30-34	Multi	M	Pfizer/BioNTech	4.8.2021	5.3.2021	5.24.2021
Donor 10	60-65	White	F	Pfizer/BioNTech	3.10.2021	3.25.2021	4.15.2021
Donor 11	55-59	White	F	Pfizer/BioNTech	3.18.2021	4.6.2021	4.26.2021
Donor 12	25-29	White	F	Pfizer/BioNTech	3.30.2021	4.16.2021	5.5.2021
Donor 13	25-29	White	M	Pfizer/BioNTech	4.5.2021	5.4.2021	5.26.2021
Donor 14	60-65	White	F	Pfizer/BioNTech	3.11.2021	3.29.2021	4.19.2021
Donor 15	25-29	White	F	Pfizer/BioNTech	3.29.2021	4.12.2021	5.3.2021
Donor 16	50-54	White	M	Pfizer/BioNTech	4.9.2021	5.4.2021	5.25.2021
Donor 17	20-24	White	F	Pfizer/BioNTech	4.5.2021	4.21.2021	5.11.2021
Donor 18	50-54	White	F	Pfizer/BioNTech	3.17.2021	4.23.2021	5.14.2021
Donor 19	25-29	White	M	Pfizer/BioNTech	3.29.2021	4.13.2021	5.3.2021
Donor 20	30-34	White	M	Pfizer/BioNTech	4.1.2021	4.23.2021	5.21.2021

Supplementary Table S1. Details of donor cohort.

	Pre-bleed						post-D1						
	well 1	well 2	well 3	well 4	Mean	SD	well 1	well 2	well 3	well 4	Mean	SD	significance for individual donors
Donor 1	1	0	0	1	0.50	0.58	8	5	4	6	5.75	1.71	p < 0.0001
Donor 2	0	0	0	0	0.00	0.00	no cells	3	5	7	5.00	2.00	p < 0.0001
Donor 3	0	0	0	0	0.00	0.00	46	45	57	51	49.75	5.50	p < 0.0001
Donor 4	1	0	0	0	0.25	0.50	27	19	29	24	24.75	4.35	p < 0.0001
Donor 5	0	0	0	0	0.00	0.00	3	6	4	6	4.75	1.50	p < 0.0001
Donor 6	1	0	0	0	0.25	0.50	5	4	1	2	3.00	1.83	p < 0.01
Donor 7	0	1	0	0	0.25	0.50	4	6	7	6	5.75	1.26	p < 0.0001
Donor 8	0	0	0	0	0.00	0.00	2	3	5	1	2.75	1.71	p < 0.001
Donor 9	2	1	1	2	1.50	0.58	81	95	83	72	82.75	9.46	p < 0.0001
Donor 10	3	1	5	4	3.25	1.71	40	62	54	45	50.25	9.74	p < 0.0001
Donor 11	1	0	0	0	0.25	0.50	3	1	2	5	2.75	1.71	p < 0.01
Donor 12	0	0	0	0	0.00	0.00	16	27	34	22	24.75	7.63	p < 0.0001
Donor 13	0	1	1	0	0.50	0.58	21	14	21	11	16.75	5.06	p < 0.0001
Donor 14	0	0	0	0	0.00	0.00	7	7	8	4	6.50	1.73	p < 0.0001
Donor 15	1	0	1	0	0.50	0.58	6	6	3	4	4.75	1.50	p < 0.001
Donor 16	0	0	1	0	0.25	0.50	26	13	24	21	21.00	5.72	p < 0.0001
Donor 17	0	0	0	0	0.00	0.00	15	7	15	9	11.50	4.12	p < 0.0001
Donor 18	0	0	0	0	0.00	0.00	12	4	9	3	7.00	4.24	p < 0.0001
Donor 19	0	0	1	2	0.75	0.96	18	21	13	16	17.00	3.37	p < 0.0001
Donor 20	0	1	0	0	0.25	0.50	2	1	2	3	2.00	0.82	p < 0.05

Supplementary Table S2. Raw SFU counts from replicate wells at the pre-bleed and post-D1 timepoints. S-antigen-specific IgG^+ SFU counts are shown for four replicate wells per donor, each seeded with 5×10^5 polyclonally stimulated PBMCs (for Donor 2 limited cell material allowed for only three replicate wells). For each donor, counts were summed across replicate wells (2×10^6 PBMCs per timepoint) and compared by an exact Poisson test, implemented as a conditional binomial test in Microsoft Excel, with the success probability set to the proportion of wells assayed at the post-D1 timepoint; p values are denoted.
